# Prevalence of chronic kidney disease after preeclampsia

**DOI:** 10.1007/s40620-016-0342-1

**Published:** 2016-08-05

**Authors:** Veronica Agatha Lopes van Balen, Julia Jeltje Spaan, Tom Cornelis, Marc Erich August Spaanderman

**Affiliations:** 1 Department of Obstetrics and Gynecology, Maastricht University Medical Center, PO Box 5800, 6202 AZ Maastricht, The Netherlands; 2 Division of Nephrology, Department of Internal Medicine, Maastricht University Medical Center, Maastricht, The Netherlands

**Keywords:** Kidney function, Preeclampsia, Postpartum, KDIGO criteria

## Abstract

**Background:**

Preeclampsia (PE), an endothelial disease that affects kidney function during pregnancy, is correlated to an increased future risk of cardiovascular and chronic kidney disease. The Kidney Disease Improving Global Outcomes (KDIGO) 2012 guideline emphasizes the combined role of glomerular filtration rate (GFR) and albuminuria in determining the frequency of monitoring of kidney function.

**Objectives:**

In this study we evaluated the prevalence of CKD in women with a history of PE. We investigated how many seemingly healthy women required monitoring of kidney function according to the KDIGO guideline.

**Methods:**

We included 775 primiparous women with a history of PE. They were at least 4 months postpartum, and had no pre-existing hypertension, diabetes or kidney disease. We estimated GFR by the CKD-Epidemiology equation and urinary albumin loss by albumin creatinine ratio in a 24-h urine collection.

**Results:**

Most women, 669 (86.3 %), had a normal GFR and absent albuminuria. Based on the KDIGO guideline, 13.7 % would require at least yearly monitoring of kidney function. Only 1.4 % were classified to be at high risk for kidney function deterioration.

**Conclusion:**

Monitoring of kidney function seems relevant for about one in seven women with a history of PE, mainly due to albuminuria. Albuminuria should be evaluated postpartum to identify those women that need further monitoring of kidney function.

**Electronic supplementary material:**

The online version of this article (doi:10.1007/s40620-016-0342-1) contains supplementary material, which is available to authorized users.

## Introduction

Preeclampsia (PE), an endothelial disease characterized by hypertension and commonly coinciding with proteinuria, is the most common cause of maternal death in the Netherlands. Maternal death due to preeclampsia occurs in 3.5 of 100,000 live births, accounting for 39 % of total maternal deaths [1]. PE affects kidney function during pregnancy and also increases the risk of future chronic hypertension, chronic kidney disease (CKD) and cardiovascular disease [2–9]. PE is associated with a fourfold increased risk of developing end-stage renal disease (ESRD) within 10 years after pregnancy [3]. Having more than one preeclamptic pregnancy, a low-birthweight offspring or a preterm delivery increases this risk even further [3].

Glomerular endotheliosis observed during a preeclamptic pregnancy may be fully reversible if it does not reach the level of glomerular scarring [10]. The reversibility is obviously dependent on cessation of the cause of endothelial injury. In the case of PE, this is effectuated by termination of pregnancy and delivery of the placenta. It can take months before the fibrinous and granular material deposits in the glomerulus fully disappear [11].

Data on follow-up of kidney function of these women are scarce. Some case–control and cohort studies suggest that most women with a history of PE do maintain good kidney function after PE [12–14]. However, there remains a group of women that will have decreased glomerular filtration rate (GFR) and/or persistent urinary protein loss [2, 5].

The existence of moderately increased albuminuria, at any glomerular filtration rate, is considered an independent risk factor for the development of cardiovascular disease [15–17]. The Kidney Disease Improving Global Outcomes (KDIGO) 2012 criteria emphasize the independent role of GFR and albuminuria in assessing the prognosis of CKD. The guideline provides us with comprehensive evidence-based indications for monitoring of patients with CKD. In this study, we determined the prevalence of CKD in a large cohort of women with a history of PE. We aimed to establish how many of these seemingly healthy former patients would qualify for further monitoring of kidney function according to the KDIGO guideline.

## Methods

In the Maastricht University Medical Center (MUMC) a postpartum cardiovascular screening is offered to women with a history of PE. The aim of this screening is to determine underlying disorders that might have contributed to the development of PE and to advise them on future pregnancies and cardiovascular implications. All women with a history of a vascular complicated pregnancy (either maternal hypertensive or fetal growth restricted) were offered a postpartum cardiovascular screening after delivery in our hospital, and some were also referred from other hospitals in the country, between 1996 and 2014. For our analysis, to improve the homogeneity of the studied population, we selected only women with PE (gestational hypertensive with de novo proteinuria) [18] who entered our program. We excluded multiparous and women who were evaluated less than 4 months after delivery. We also excluded women diagnosed with hypertension, diabetes mellitus or kidney disease prior to the complicated pregnancy. All measurements were performed in our assessment unit at the Maastricht University Medical Center under controlled circumstances.

### Measurements

Measurements during the postpartum cardiovascular screening were performed in standardized environmental conditions. Blood pressure was recorded for a period of 30 min at 3-min intervals using a semiautomatic oscillometric device in half-sitting position. Median values of nine subsequent recordings were used for analysis. An extensive (family) history was taken. Urine analysis was done to determine albumin loss on a urine sample collected over 24 h. Albuminuria was measured by immunochemical analysis. We categorized women into normal to mild albuminuria <3 mg/mmol, moderately increased albuminuria 3–30 mg/mmol, and severely increased albuminuria >30 mg/mmol [19]. We also collected blood samples for serum creatinine, which was obtained by the Jaffe method until 2012 and thereafter through an enzymatic method. All three measurements were performed with Cobas 8000 modular analyzer series (Roche Diagnostics, Basel, Switzerland).

We estimated GFR by the CKD-Epidemiology (CKD-EPI) equation in ml/min/1.73 m^2^. Both formulas are dependent on serum creatinine and used for females and Caucasians. When serum creatinine was below or equal to 0.7 mg/dl, we used 144 × (serum creatinine/0.7)^−0.329^ × 0.993^age^. When serum creatinine was above 0.7 mg/dl we used 144 × (serum creatinine/0.7)^−1.209^ × 0.993^age^. Albuminuria was quantified by the albumin creatinine ratio (ACR).

### KDIGO

The KDIGO guideline provides a table with recommendations on frequency of monitoring of kidney function based on the risk of progression of CKD, and another table on monitoring by a general clinician or referral to a nephrologist. We combined both tables into one overview table (Table 1) [19]. In this table the yellow color indicates a preferred frequency of monitoring once a year, whereas orange indicates monitoring twice a year, light red three times a year, and dark red four times a year or more. Referral for monitoring of kidney function by more specialized personnel, preferably a nephrologist, is indicated for severely increased albuminuria (ACR > 30) or decreased GFR (<30), according to the KDIGO guideline, as is shown.

**Table 1:**
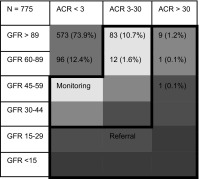
KDIGO table on frequency of monitoring showing the prevalence of chronic kidney disease in primiparous women with a history of preeclampsia

Frequency of monitoring based on the risk of progression of CKD depending on ACR and GFR [19]. Once-yearly monitoring is represented by yellow, twice-yearly by orange, three times per year by light red, and four times per year by dark red. GFR is expressed in ml/min/1.73 m^2^ and ACR in mg/mmol. Referral indicates the need for referral to more specialized care, preferably by a nephrologist

*KDIGO* Kidney Disease Improving Global Outcomes, *GFR* glomerular filtration rate, *ACR* albumin creatinine ratio

The KDIGO table on cardiovascular mortality is based on a meta-analysis [20] of 105,872 individuals, extracted from 14 different studies (see Table S1). To calculate the adjusted relative risk of cardiovascular mortality for our study group we classified all women based on this table.

### Statistical analysis

All analyses were performed using SPSS version 21.0, property of IBM (SPSS, Chicago, IL, USA) and supplied by Maastricht University. Data were expressed as group means and standard deviation or medians and interquartile ranges (IQR). A p value <0.05 was considered to indicate a statistically significant difference. To analyze the differences between groups with normally distributed data we used the unpaired t test. For non-normal distributed data, we used the Mann–Whitney U test. Dichotomous data were analyzed by Chi square test. We corrected for months postpartum, age, gestational onset of hypertensive complication and gestational age at delivery where appropriate.

## Results

**Fig. 1:**
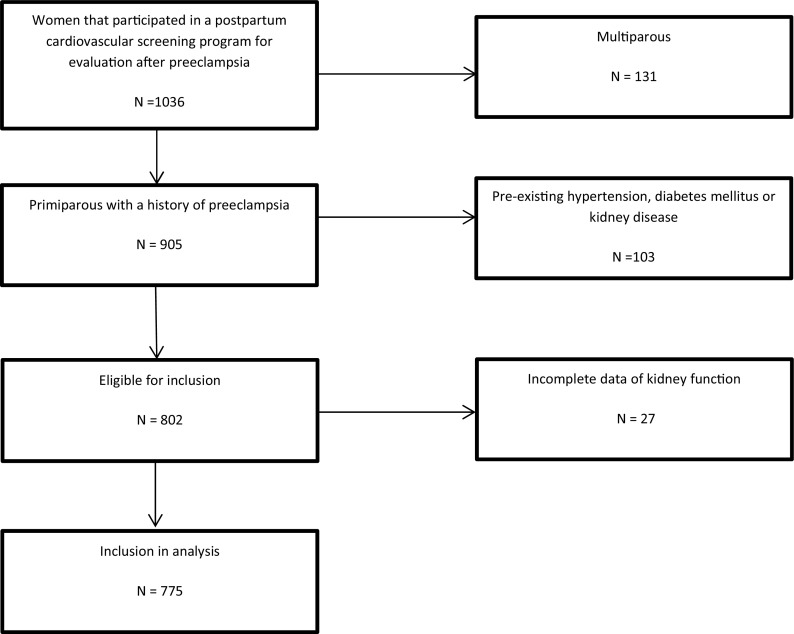
Flowchart

Women with a history of PE, at least 4 months postpartum, were selected after exclusion of those that were multiparous or had pre-existing hypertension, diabetes mellitus and/or kidney disease (Fig. 1). Data required for categorization of kidney function were incomplete in 3.1 % of cases, leaving 775 complete cases.

Table 2 shows the characteristics of our study group. Women were on average 10 months postpartum, normotensive, had an early onset PE (<34 weeks gestation) and gave birth prematurely. The baseline values were equal to those of women excluded due to missing urinary analyses. Women with persistent moderate to severely increased albuminuria (ACR > 3 mg/mmol) were younger, gave birth at an earlier gestational age to smaller children and had higher blood pressure compared to women with normal to mildly increased albuminuria (ACR < 3 mg/mmol). They had an average amount of protein loss of 196 mg/24 h [IQR 154–237] and used significantly more antihypertensive drugs, in particular renin-angiotensin system (RAS) blocking drugs. Based on the KDIGO guideline on the frequency of monitoring (Table 1), for 106 (13.7 %) women with a history of PE, monitoring of kidney function was indicated once yearly (yellow category). This was mostly due to their having moderate-to-severely increased ACR with a normal GFR (>90) (11.9 %). Only 1.8 % had a decreased GFR (<90) and moderate-to-severely increased ACR. In addition, 11 (1.4 %) women resulted as needing more specialized care, preferably by a nephrologist, based on severely increased albuminuria (ACR > 30 mg/mmol). Table 3 details the postpartum renal albumin loss categorized by time interval. Moderate to increased albumin loss was most frequent within the first months after delivery and was less prominent in women who were more than 1 ½ years postpartum. Women who were 2 years postpartum had an increased ACR in 6 % of cases. Table S1 (supplementary appendix) presents the KDIGO table on calculated cardiovascular mortality risk for our study group. It shows that about 25 % of women had a relative risk of 0.9 of cardiovascular mortality, 22 % a 1.3 relative risk, and 10 % a 1.5 relative risk. Some women (7.6 %) even had a 2.3 increased risk. The median adjusted relative risk of cardiovascular mortality was 1.25 ± 0.6 (not shown in Table S1). The increased cardiovascular mortality risk was mostly based on increased albumin loss as opposed to a reduced GFR.

**Table 2:** Characteristics of all primiparous women with a history of preeclampsia and stratified by albumin creatinine ratio (ACR)

	All participants n = 775	ACR < 3 mg/mmol n = 669	ACR ≥ 3 mg/mmol N = 106	p value
Age (years)	31 ± 4.2	31 ± 4.1	30 ± 4.5*	<0.01
Months postpartum	10 [6–18]	12 [7–19]	10 [5–10]	0.945
BMI (kg/m^2^)	25.0 ± 4.8	25 ± 4.6	25 ± 5.2	0.872
Smoking (%)	109 (14 %)	94 (14 %)	15 (14 %)	0.978
Gestational age at diagnosis (weeks)	31^+ 5^ ± 4^+ 1^	31^+ 6^ ± 4^+ 1^	30^+ 6^ ± 4^+ 0^ *	0.049
Gestational age at delivery (weeks)	33^+3^ ± 3^+5^	33^+ 4^ ± 3^+ 5^	32^+ 5^ ± 3^+ 6^ *	0.012
Birth weight (g)	1856 [1188–2447]	1890 [1230–2450]	1657 [980–2266]*	0.010
Birth weight percentile	20 [9–40]	20 [9–45]	18 [6–36]*	0.037
Systolic blood pressure (mmHg)^†^	116 ± 13	116 ± 12	120 ± 12*	<0.01
Diastolic blood pressure (mmHg)^†^	72 ± 9	71 ± 9	75 ± 10*	<0.01
Glomerular filtration rate (ml/min/1.73 m^2^)^†^	105 ± 15	105 ± 15	107 ± 16	0.471
Antihypertensive drugs	66 (8.5 %)	52 (7.7 %)	14 (13.2 %)*	0.031
ACR mg/mmol	0.9 [0.5–1.9]	0.8 [0.5–1.3]	5 [4–10]	
RAS blocking drugs (%)	26 (3.4 %)	16 (2.4 %)	10 (9.4 %)*	<0.01

Data were are expressed as group means and standard deviation or median and interquartile range

*BMI* body mass index, *RAS* renin–angiotensin system. For other abbreviations, see previous tables

* p < 0.05

^+^Adjusted for months postpartum

^†^Adjusted for gestational age at delivery and age

**Table 3:** Albuminuria after preeclampsia categorized by time interval

Months postpartum	>3 ACR mg/mmol n/total (%)	Adjusted odds
4–5	51/150 (25 %)	Ref
6–11	33/248 (12 %)	0.43 (CI 0.26–0.71)*
12–17	13/96 (12 %)	0.44 (CI 0.23–0.87)*
18–23	1/58 (2 %)	0.06 (CI 0.00–0.46)*
>24	8/117 (6 %)	0.25 (CI 0.11–0.57)*

Odds ratio represents the risk of persistent albuminuria (>3 ACR) at a certain time period postpartum compared with to the reference group (4–6 months postpartum), corrected for gestational age at delivery and age. An asterisk represents a p value less than <0.05

## Discussion

Our study indicates that, based on the KDIGO monitoring guideline, about one in seven women after PE should have at least yearly monitoring of their kidney function, this being mainly due to a moderately increased albuminuria. Only 1.4 % were classified as at high risk for kidney function deterioration and in need of referral to specialist care, preferably a nephrologist. About 6 % of women had an increased ACR 2 years postpartum.

PE can manifest as a combination of maternal and fetal factors. It can be considered as more than one disease with major differences between near-term mild PE and PE that is associated with low birthweight and preterm delivery [21]. Our data indicate that mainly persistent albuminuria would classify 13.7 % women after PE as having CKD. The prevalence of CKD in the general adult, and usually older, population is approximately 10 % [22]. Women with a history of PE are younger and have a four to fivefold increased risk of ESRD compared to women with no history of PE [3]. Having more than one preeclamptic pregnancy, a low-birthweight offspring or a preterm delivery increases the risk of CKD even further [3].

The KDIGO guideline is written for the general population and indicates that kidney disease persisting for more than 4 months is considered to be chronic, irrespective of the etiology. This provides us with guidance on how often we should follow women with a history of PE after delivery to ensure that albuminuria will regress over time and to clarify which women should be further investigated for underlying kidney disease. The persistence of albuminuria is considered to be due to either an undiagnosed pre-existing kidney disease, or a higher susceptibility of these women to develop CKD, or irreversible kidney damage during the preeclamptic pregnancy. Though kidney disease is known in some cases to be entirely reversible, either spontaneously or with treatment, it is prudent to ascertain whether this actually happens [23].

The prevalence of albuminuria in our study population is lower compared to other data that mention 42 % after 3–5 years postpartum and 20–30 % after 7 years postpartum [5, 12, 24, 25]. On the contrary, some other studies conducted at a later postpartum interval show no increased persistent (micro)albuminuria [2, 14]. We did, however, exclude women with known conditions that affect kidney function. It could be that, due to the homogeneity of the disease of PE, kidney function is affected differently, depending on the severity of the disease, but also recovers at a different time rate, possibly depending on the woman’s general health. Another contributing factor could be the method used for the acquisition of data on albuminuria as some studies use 24-h and others 8-h collection or a single urine sample.

We would also like to address the notion that the risk of cardiovascular mortality is increased in women with a history of PE. As the risk of cardiovascular disease is also increased in women with CKD [26], we could hypothesize that women with a history of PE and CKD have an even more increased risk of cardiovascular disease. Appropriate treatment aimed at preventing progression of cardiovascular disease is important since blood pressure control, lifestyle advice and exercise can slow down or even reverse progression of both CVD and CKD [21]. Appropriate blood pressure regulation seems particularly relevant for this group, as in our study women with persistent albuminuria had a higher blood pressure compared to those with normal to mildly increased albuminuria. They did already use significantly more antihypertensive medication, in particular RAS blocking drugs. Blood pressure regulation, as part of cardiovascular risk management, improves the prognosis of CKD [27]. Albuminuria screening seems not only effective but also cost-effective, when taking possible treatment with angiotensin-converting-enzyme (ACE) inhibitors into account, as it reduces the risk of both CKD and cardiovascular disease [28]. Interestingly, screening of albuminuria in a high-risk population is found to correlate with the occurrence of future renal replacement therapy [29]. This makes screening for albuminuria in a population of women with a history of PE even more plausible. From a practical point of view, we would advise evaluation of kidney function, for women with a history of PE, at the regular 6-weeks postpartum control. Women who then have microalbuminuria could be seen 1 year thereafter by their general physician for follow-up of kidney function. If a cardiovascular screening should be available the inclusion of a test for microalbuminuria would be advisable.

There are some shortcomings of our study that limit extrapolation to other populations. First of all, even though the KDIGO offers a guideline with evidence-based decisions that provides the possibility to take both GFR and ACR into account when evaluating kidney function, it is difficult to extrapolate outcomes and the need for frequency of follow-up for young and otherwise healthy women from the CKD tables of KDIGO. It is possible that these women will have very different relative risk changes and rates of progression than corresponding individuals with other renal diseases like diabetes or primary glomerular disease. The KDIGO guideline does not define patients by etiology. Secondly, it may be that our population does not completely represent the general formerly preeclamptic group of women as our population primarily consists of women with early onset PE. Early onset disease may cause an overestimation of the prevalence of CKD due to the severity of the disease and its impact on the endothelium. On the other hand, the results might underestimate the prevalence of CKD after PE due to the exclusion of comorbidities known to be related to kidney dysfunction, such as diabetes mellitus, pre-existing hypertension and known kidney disease. The cross-sectional nature of this study is also an inevitable shortcoming, as is the lack of a control group. Because not all women with a history of PE that delivered in our hospital partook in the screening and because women were referred from other hospitals we are not able to fully generalize to the overall PE population. A future longitudinal study is needed to ascertain changes over time, preferably including data on pre-pregnancy GFR or albuminuria values to determine a possible pre-existing kidney condition.

The key strengths of this study are the relatively long postpartum period, when compared to a regular checkup at 6 weeks after delivery and the large study population. Recent studies indicate that CKD-EPI gives the best estimate of GFR when compared to the gold standard of I^125^ iothalamate [30]. It has the highest accuracy in women when compared to both the MDRD and Cockcroft-Gault formula [31] and the use of CKD-EPI has, to date, not been reported in women with a history of PE.

## Conclusion

Monitoring of kidney function seems relevant for about one in seven formerly preeclamptic women, mainly due to albuminuria. Albuminuria should be evaluated postpartum to identify those women that need further monitoring of kidney function.

## Electronic supplementary material

Below is the link to the electronic supplementary material.

Supplementary material 1 (DOCX 19 kb)
