# Supplementary material for: Prevalence of chronic kidney disease after preeclampsia
Source: J Nephrol. 2016 Aug 5;30(3):403–9. doi: 10.1007/s40620-016-0342-1 (PMC5437128; doi:10.1007/s40620-016-0342-1)
Supplement: Supplementary file 1 — Supplementary material 1 (DOCX 19 kb) [file 40620_2016_342_MOESM1_ESM.docx]

**Appendix**

**Table 5: KDIGO table on cardiovascular mortality risk showing the number of primiparous women with a history of preeclampsia in each risk category**

| **N = 775** | **ACR <1** | | **ACR 1-2,9** | | **ACR 3-29,9** | | **ACR > 30** | |
| --- | --- | --- | --- | --- | --- | --- | --- | --- |
| **eGFR >105** | **0.9** | **193 (24.9%)** | **1.3** | **152 (19.6%)** | **2.3** | **58 (7.5%)** | **2.0** | **9 (1.2%)** |
| **eGFR 90-105** | **Ref** | **143 (18.5%)** | **1.5** | **85 (11.0%)** | **1.7** | **25 (3.2%)** | **3.7** | **-** |
| **eGFR 75-90** | **1.0** | **60 (7.7%)** | **1.3** | **24 (3.1%)** | **1.6** | **6 (0.7%)** | **3.7** | **1 (0.1%)** |
| **eGFR 60-75** | **1.1** | **9 (1.2%)** | **1.4** | **3 (0.4%)** | **2.0** | **6 (0.8%)** | **4.1** | **-** |
| **eGFR 45-60** | **1.5** | **-** | **2.2** | **-** | **2.8** | **-** | **4.1** | **1 (0.1%)** |
| **eGFR 30-45** | **2.2** | **-** | **2.7** | **-** | **3.4** | **-** | **5.2** | **-** |
| **eGFR 15-30** | **1.5** | **-** | **7.9** | **-** | **4.8** | **-** | **8.1** | **-** |

The estimated cardiovascular mortality risk is based on a meta-analysis of 105,872 people. Each cell represents a pooled relative risk from this meta-analysis. Colors reflect the ranking of adjusted relative risk. The point estimates for each cell were ranked from 1 (lowest) to 28 (highest). The categories with rank numbers 1–8 are green, rank numbers 9–14 are yellow, rank numbers 15–21 are orange, and the rank numbers 22–28 are colored red (32). Normal to mild albuminuria is further categorized into ACR <1 and 1-3 mg/mmol. Number of women in each cell with percentage is given. GFR is expressed in ml/min/1.73 m^2^ and ACR in mg/mmol.
